# Large Versus Small Gastric Pouch for Roux-en-Y Gastric Bypass in Individuals With Type 2 Diabetes and a Body Mass Index < 35 kg/m2: Six-Year Outcomes

**DOI:** 10.3389/fendo.2022.913062

**Published:** 2022-09-01

**Authors:** Xiang Gao, Song Dai, Guohui Wang, Weizheng Li, Zhi Song, Zhihong Su, Shaihong Zhu, Liyong Zhu, Pengzhou Li

**Affiliations:** Department of General Surgery, Third Xiangya Hospital, Central South University, Changsha, China

**Keywords:** gastric pouch, type 2 diabetes, obesity, Roux-en-Y gastric bypass, marginal ulcer

## Abstract

**Background:**

Roux-en-Y gastric bypass (RYGB) results in extraordinary weight loss and glycemic control outcomes for patients with obesity; however, the effect of gastric pouch size is still unclear, and the reported results are contradictory. Additionally, long-term data on type 2 diabetes (T2D) patients with low body mass index (BMI) are sparse. This study was to assess the effect of 6-year outcomes in Chinese patients with T2D and a BMI < 35 kg/m2 who underwent RYGB with gastric pouches of different sizes.

**Methods:**

A retrospective cohort study was performed. There were 42 patients in the large gastric pouch group (L) and 53 patients in the small gastric pouch group (S). Baseline demographic history, pre- and postoperative BMI, waist circumference, and glucose- and lipid metabolism-related indicators were compared.

**Results:**

Assessments were completed in 100%, 100%, 93.6%, and 89.4% of patients at baseline, 1 year, 3 years, and 6 years, respectively. At 6 years, the changes in BMI and fasting plasma glucose were greater in the S group (-4.25 ± 0.51 kg/m2 and -4.58 ± 0.73 mmol/l) than in the L group (-2.06 ± 0.48 kg/m2 and -2.64 ± 0.61 mmol/l). The independent predictors of complete remission of T2D were preoperative BMI and the size of the gastric pouch. A large gastric pouch was associated with a higher risk for marginal ulcers.

**Conclusions:**

A small gastric pouch results in better weight loss and glycemic control. High preoperative BMI and a small gastric pouch are associated with better T2D remission rates. A large gastric pouch leads to a higher incidence of marginal ulcers.

## Introduction

In 1967, Mason completed the world’s first gastric bypass surgery (1). After over 60 years of development and improvement, laparoscopic gastric bypass surgery has become a classic bariatric surgery method (2). Roux-en-Y gastric bypass (RYGB) is currently considered to be a safe and effective treatment that can successfully achieve glycemic control and weight loss and has been widely performed worldwide (3, 4). In 2004, Wang CC completed the first RYGB surgery in mainland China. There was a gap in the understanding of surgical mechanisms and surgical techniques in the early stage. Additionally, patients with type 2 diabetes (T2D) in East Asia generally have a lower body mass index (BMI) than those in Europe and the United States (5, 6). In China, the pathological characteristics of T2D are mainly a BMI < 35 kg/m2, central obesity, early islet cell failure and insulin resistance (7, 8). All patients hope to achieve better glycemic control or T2D remission, not weight loss.

Based on the characteristics of these patients, we initially performed RYGB using a large gastric pouch and a short Roux limb and biliopancreatic limb. Over time, we found that some of the patients had relatively poor glycemic control and a high incidence of postoperative ulcers. Based on improvements in surgical knowledge and technology, we performed subsequent gastric bypass surgeries using a small gastric pouch. Controversy regarding the size of the gastric pouch has persisted over the past 30 years. Scholars have performed many studies on the postoperative efficacy of different gastric pouch sizes (9–11). Much of the recent literature focuses on the short-term impact of gastric pouch size on the weight loss of patients with BMI > 35 kg/m2 after RYGB, and the results are controversial (12–14). However, long-term data on metabolism-related indicators, especially from Chinese T2D patients with a low BMI (< 35 kg/m2), are rarely reported.

Thus, we performed this retrospective study to compare the long-term efficacy of RYGB with different gastric pouch sizes in Chinese T2D populations with a BMI < 35 kg/m2.

## Methods

**Figure 1:**
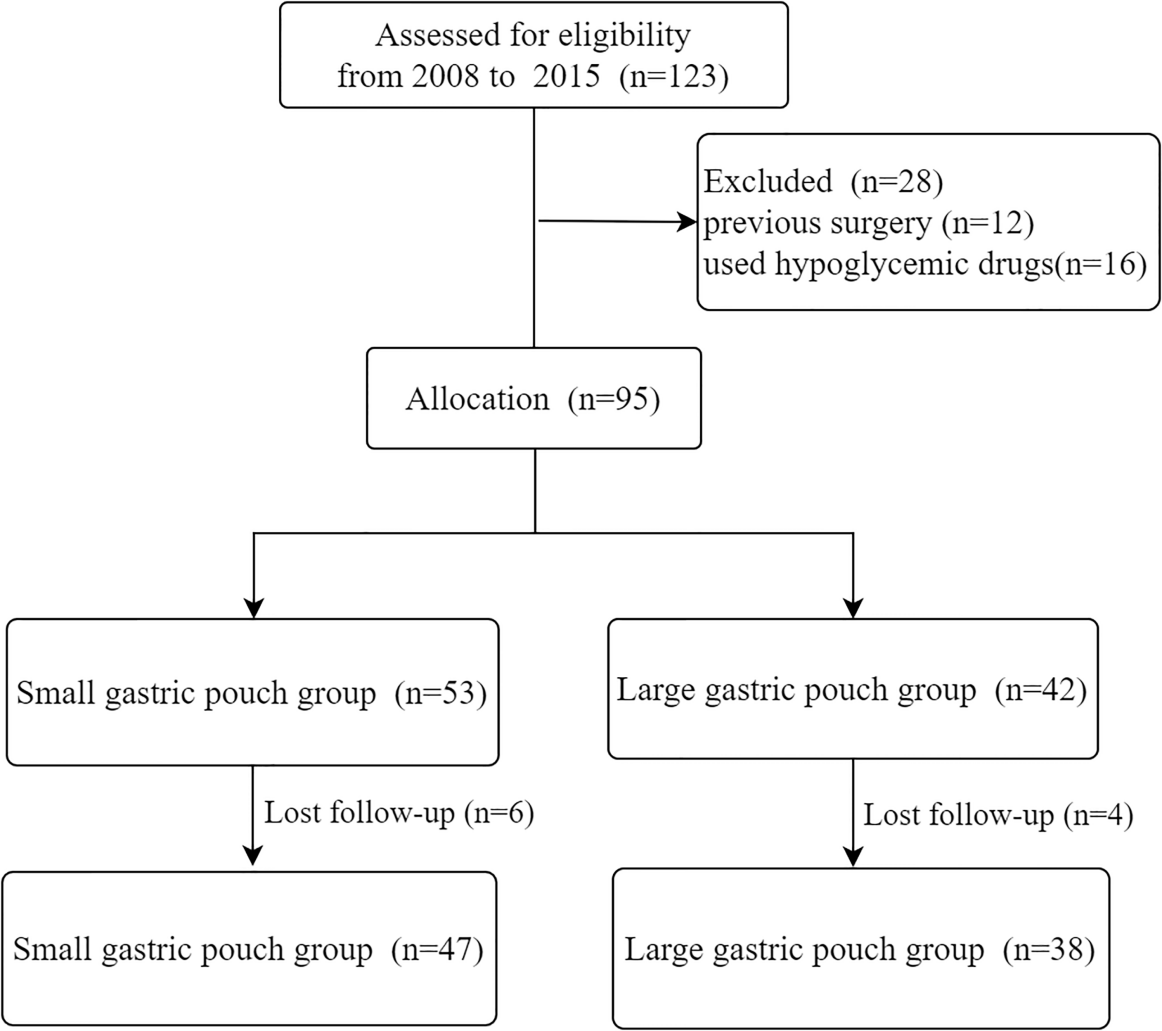
A flow chart of the eligible patients included in this study.

The study protocol was approved by the local institutional review board. In brief, the study was a two-group, retrospective, comparative study of RYGB in 95 patients, in which the effects of a small gastric pouch were compared with those of a large gastric pouch. The eligibility criteria were as follows: a diagnosis of T2D based on the 1999 World Health Organization standard (15), age between 18 years and 65 years, BMI ≤35 kg/m2, and better islet cell function (in the oral glucose tolerance test, the peak value of C-peptide was twice the base value). The exclusion criteria were as follows: drug or alcohol dependence, psychiatric illness or inability to complete follow-up, preoperative gastroscopy suggesting an active ulcer, and Helicobacter pylori infection detected by the carbon 13 breath test. Informed consent was obtained from all individual participants included in the study. All of the surgeries were performed by the same surgical team at our center. Ninety-five patients (53 patients in the small gastric pouch group and 42 patients in the large gastric pouch group) were included in this study from 2008 to 2015. Overall, the 6-year follow-up rate was 89.5%; hence, 85 of the 95 patients were evaluated to assess the efficacy and safety of RYGB with large and small gastric pouch sizes (38 and 47, respectively) (**Figure 1**).

### Surgical Procedure

**Figure 2:**
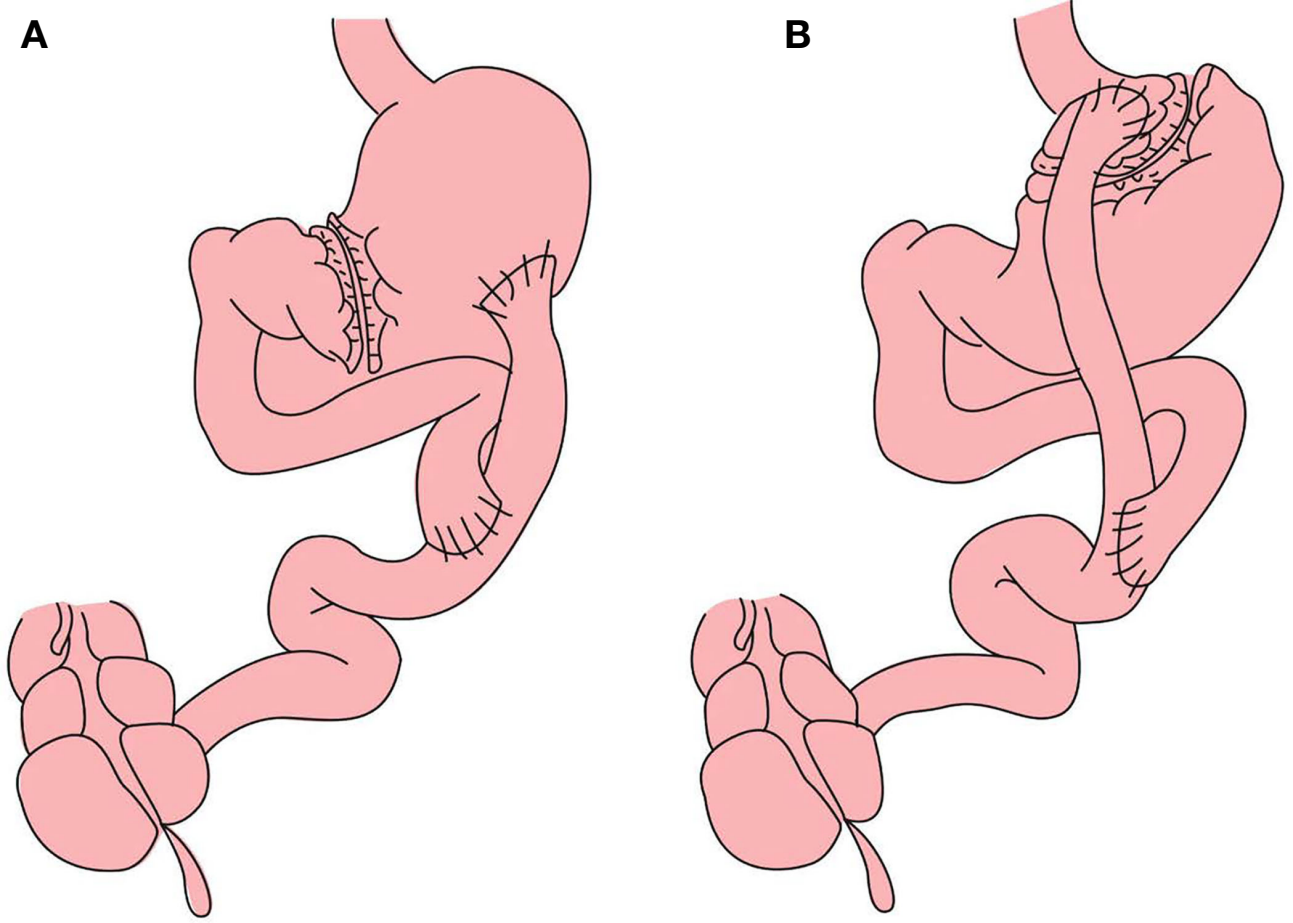
**(A)** Roux-en-Y gastric bypass with large gastric pouch): The Roux limb from the anastomoses of the stomach and distal jejunum to the second anastomosis of the proximal jejunum and distal jejunum is 50 cm. The biliopancreatic limb from the ligament of Treitz to the second anastomosis of the proximal jejunum and distal jejunum is 50 cm **(B)** RYGB with small gastric pouch: The length of alimentary limb and biliopancreatic limb is 50 cm respectively.

RYGB with a large gastric pouch has been described in a previous study (16). In brief, the subtotal stomach (beginning at the greater curvature and ending at the angular incisure, >50 ml) and the jejunum, 50 cm distal from the ligament of Treitz, are dissociated. An end-to-side anastomosis connects the distal jejunum to the posterior wall of the stomach. This operation maintains a large gastric volume. The Roux limb from the anastomoses of the stomach and distal jejunum to the second anastomosis of the proximal jejunum and distal jejunum is 50 cm. The biliopancreatic limb from the ligament of Treitz to the second anastomosis of the proximal jejunum and distal jejunum is 50 cm. RYGB with a small gastric pouch (approximately 10-20 mL, with the rest of the stomach excluded) is based on the lesser curvature and ends at the angle of His, and the Roux and biliopancreatic limbs are 50 cm in length (**Figure 2**). In both procedures, the Roux limb was anastomosed to the gastric pouch in an antecolic fashion. During surgery, the surgeon used a linear stapler to perform the gastric pouch and measured the width and height of gastric pouch by scale of linear stapler. The gastric pouch volume was calculated by the formula: volume= (width×width×height)÷π.

### Collection of Clinical Indicators and Assessment

All patients were routinely administered proton pump inhibitors and multivitamins after surgery. For long-term follow-up, All patients received the same dietary guidance and rehabilitation exercises after surgery, and all data collection and verification were managed by a dedicated person. We collected clinical information, including BMI, waist circumference, high-density lipoprotein (HDL), low-density lipoprotein (LDL), total cholesterol (TC), triglyceride (TG), glycosylated hemoglobin (GHbA1c), and fasting plasma glucose (FPG), for every patient. The percent excess weight loss (%EWL) was calculated with the following formula: %EWL = [(initial weight − Postoperative weight)/(initial weight − ideal weight)] × 100. The ideal BMI is 24kg/m2 in Chinese patients according WHO guidelines. The comorbidities of interest in the present study were bleeding, gallstone diseases, intestinal obstruction, marginal ulcer, dumping syndrome and anemia. Diabetes complete remission was defined as an HbA1c level < 6% and/or an FPG level<5.6 mmol/l and 12 months without active pharmacological intervention (17). Homeostatic model assessment insulin resistance (HOMA-IR) was calculated with the following formula: (FPG×FINS)/22.5.

### Statistical Analysis

All continuous variables conforming to a normal distribution were described as the mean ± standard deviation (SD), and percentages and frequencies are used to describe categorical variables. Normality of the data was verified using the Kolmogorov–Smirnov test. Statistical significance was indicated by P <0.05, and a paired t test was used to compare different time points within the group. Independent samples t tests were used for comparisons between the large gastric pouch group and the small gastric pouch group at 6 years postsurgery. The chi-square test was used for categorical variables. Analysis of variance was used to evaluate the differences in the two groups at postsurgery. Logistic regression analysis was applied to explore factors associated with T2D remission and marginal ulcers. SPSS version 26.0 (SPSS IBM, USA) was used to perform the statistical analyses.

## Results

### Study Cohort

The baseline characteristics of the 95 patients who underwent different surgeries are shown in **Table 1**. The patients in the small gastric pouch group, compared with those in the large gastric pouch group, were older on average (48.62 ± 8.27 vs. 47.00 ± 7.90) and had a higher average BMI (30.09 ± 1.66 kg/m2 vs. 29.39 ± 1.87 kg/m2); GHbA1c, TG, TC, HDL, LDL, HOMA-IR, waist circumference, duration of diabetes, smoking and metabolic syndrome data were collected. There was no significant difference between the two groups at baseline.

**Table 1:** Characteristics of the patients at baseline.

	Large gastric pouch group	Small gastric pouch group	P
Number	42	53	
Age (yr)	47.00±7.90	48.62±8.27	0.336
Sex (% male)	28 (66.7%)	33 (62.2%)	0.657
BMI (kg/m^2^)	29.39±1.87	30.09±1.66	0.057
Waist circumference (cm)	102.18±3.62	103.31±3.20	0.110
Duration of diabetes	6.32±1.27	6.81±1.39	0.080
Smoking (%)	6 (14.3%)	5 (9.4%)	0.681
Fasting plasma glucose (mmol/l)	9.79±1.98	10.53±2.23	0.095
GHbA1c (%)	8.68±1.64	9.29±1.75	0.086
Metabolic syndrome (%)	26 (61.9%)	29 (54.7%)	0.481
TG (mmol/l)	3.31±0.49	3.28±0.61	0.796
TC (mmol/l)	5.08±0.99	4.69±1.10	0.076
LDL (mmol/l)	2.56±0.75	2.41±0.90	0.388
HDL (mmol/l)	1.38±0.25	1.31±0.31	0.238
HOMA-IR	8.67±1.91	9.33±1.68	0.077

BMI, body mass index; GHbA1c, glycated haemoglobin; TG, triglyceride; TC, total cholesterol; HDL, high-density lipoprotein; LDL, low-density lipoprotein; HOMA-IR, homeostasis model assessment insulin resistance

### Weight Loss and Lipid Profile

**Figure 3:**
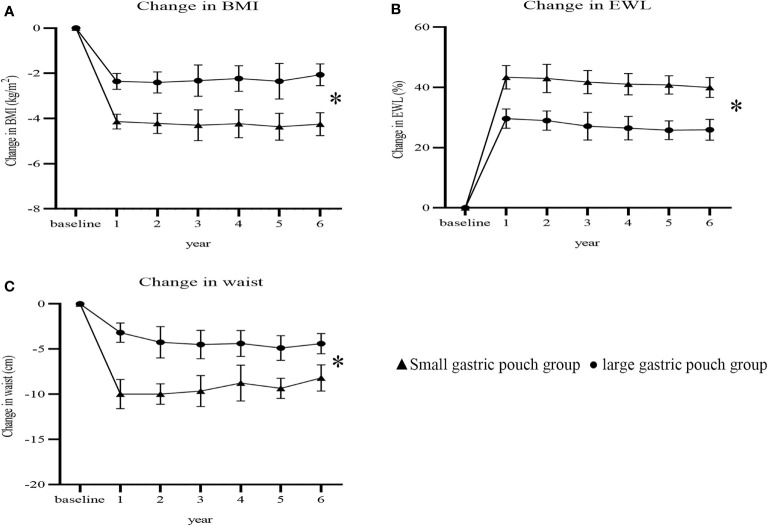
Changes in BMI, %EWL and waist circumference after surgery between the two groups (P=0.021, P=0.038 and P=0.018). Change in BMI **(A)**; change in %EWL **(B)**; change in waist **(C)**. BMI, body mass index; %EWL, percent excess weight loss. * P<0.05 for comparisons of postoperative changes between the small gastric pouch group and large gastric pouch group using 2-factor mixed analysis of variance.

BMI, waist circumference and lipid profiles at 6 years postsurgery are presented in **Table 2**. At 6 years postoperation, the BMI of the small gastric pouch group and large gastric pouch group dropped from 30.09 ± 1.66 kg/m2 and 29.39 ± 1.87 kg/m2 to 25.87 ± 1.35 kg/m2 and 26.71 ± 1.21, respectively (P=0.004). The waist measurements of the small gastric pouch group and the large gastric pouch group dropped from 103.31 ± 3.20 cm and 102.18 ± 3.62 cm to 95.04 ± 3.56 cm and 96.83 ± 4.04 (P=0.033), respectively. As **Figure 3** shows, there were significant differences (P = 0.021, P=0.038, P = 0.018) in the changes in BMI, %EWL and waist circumference between the two groups. However, no significant difference in HDL, LDL, TG or TC levels was found between the two groups at 6 years postsurgery (P=0.106, P=0.592, P=0.334 and P= 0.377, respectively).

**Table 2:** Metabolic and weight loss changes from baseline at 6 years.

	Large gastric pouch group	Small gastric pouch group	P
N	38	47	
BMI (kg/m^2^)			
6 years	26.71±1.21	25.87±1.35	0.004
Change from baseline	-2.06±0.48	-4.25±0.51	0.000
Waist circumference (cm)			
6 years	96.83±4.04	95.04±3.56	0.033
Change from baseline	-4.40±1.12	-8.19±1.46	0.000
FPG (mmol/l)			
6 years	6.25±1.38	5.51±1.21	0.010
Change from baseline	-2.64±0.61	-4.58±0.73	0.000
GHbA1c (%)			
6 years	7.81±0.77	6.10±0.96	0.000
Change from baseline	-0.71±0.39	-2.36±0.41	0.000
TG (mmol/l)			
6 years	2.17±1.40	2.48±1.51	0.334
Change from baseline	-0.33±1.15	-0.87±1.32	0.051
TC (mmol/l)			
6 years	4.28±1.04	4.09±0.93	0.377
Change from baseline	-0.51±1.09	-0.82±0.86	0.146
LDL (mmol/l)			
6 years	2.12±0.68	2.05±0.52	0.592
Change from baseline	-0.21±0.57	-0.40±0.44	0.087
HDL (mmol/l)			
6 years	1.16±0.35	1.31±0.47	0.106
Change from baseline	0.12±0.35	0.19±0.36	0.370
HOMA-IR			
6 years	5.06±1.36	2.43±1.13	0.000
Change from baseline	-2.83±1.24	-6.49±1.09	0.000

BMI, body mass index; FPG, fasting plasma glucose GHbA1c, glycated haemoglobin; TG, triglyceride; TC, total cholesterol; HDL, high-density lipoprotein; LDL, low-density lipoprotein; HOMA-IR, homeostasis model assessment insulin resistance.

### Glycemic Control

**Figure 4:**
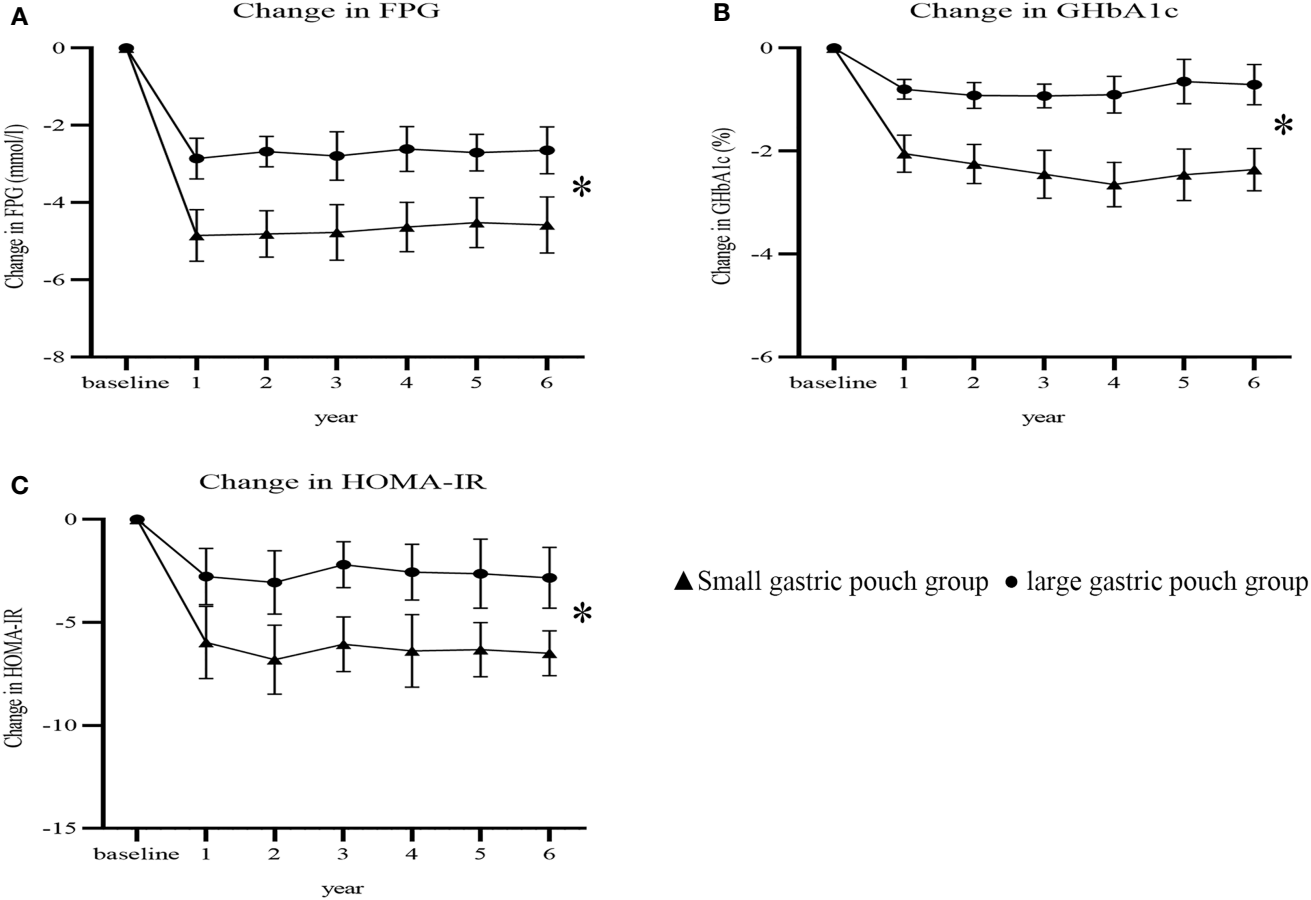
Changes in FPG, GHbA1c and HOMA-IR after surgery between the two groups (P=0.035, P=0.017 and P=0.000). Change in FPG **(A)**; change in GHbA1c **(B)**; change in HOMA-IR **(C)**. FPG, fasting plasma glucose; GHbA1c, glycated hemoglobin; HOMA-IR, homeostasis model assessment insulin resistance. * P<0.05 for comparisons of postoperative changes between the small gastric pouch group and large gastric pouch group using 2-factor mixed analysis of variance.

At 6 years postsurgery, the FPG, GHbA1c and HOMA-IR values in the small gastric pouch group were 5.51 ± 1.21 mmol/l, 6.10 ± 0.96% and 2.43 ± 1.13, respectively, while in the large gastric pouch group, these values were 6.25 ± 1.38 mmol/l, 7.81 ± 0.77% and 5.06 ± 1.36 (P=0.010, P=0.000 and P=0.000, respectively); significant differences between the two groups were evident (**Table 2**). The decreases from baseline in FPG, GHbA1c and HOMA-IR were greater in the small gastric pouch group than in the large gastric pouch group (P=0.035, P=0.017 and P=0.000, respectively) (**Figure 4**). The T2D complete remission rate of the small gastric pouch group was significantly higher than that of the large gastric pouch group at 1, 3, and 6 years postsurgery (P=0.024, P=0.039 and P=0.028, respectively) (**Table 3**). The regression analysis of the T2D complete remission rate is shown in **Table 4**. Preoperative BMI and the size of the gastric pouch were significant factors for predicting T2D complete remission at 6 years postsurgery (P=0.04 and P=0.01). Each increase of 1 kg/m2 in the preoperative BMI increased the relative risk for T2D complete remission by 8% (95% CI 5–11%). The relative risk for T2D complete remission after RYGB with a small gastric pouch was 1.11 (95% CI 1.07-1.16) compared to RYGB with a large gastric pouch. Other variables, including sex, age at surgery, duration of T2D and HOMA-IR, did not predict complete remission of T2D (P >0.05).

**Table 3:** The complete remission rate of T2DM at 1, 3 and 6 years.

	1-year (N=95)	3-year (N=89)	6-year (N=85)
Small gastric pouch	30 (56.6%)	24 (49.0%)	22 (46.8%)
Large gastric pouch	14 (33.3%)	11 (27.5%)	9 (23.7%)
P	0.024	0.039	0.028

**Table 4:** Multivariate logistic regression analysis indicating correlations between variables and T2DM remission and marginal ulcers at 6 years postsurgery.

	T2D remission		Marginal ulcer	
	P	Odds ratio with 95% CI	P	Odds ratio with 95% CI
Male gender	0.23	0.98 (0.87-1.15)	0.30	0.98 (0.72-1.12)
Age at surgery (years)	0.07	0.58 (0.37-1.07)	0.16	1.75 (0.47-1.94)
Duration of T2D (years)	0.16	0.87 (0.62-2.91)	0.08	1.62 (0.59-2.31)
Preoperative BMI (kg/m^2^)	0.04	1.08 (1.05-1.11)	0.55	0.58 (0.51-1.34)
HOMA-IR	0.43	0.79 (0.71-1.04)	0.63	0.66 (0.57-1.26)
Size of gastric pouch	0.01	1.11 (1.07-1.16)	0.04	1.13 (1.08-1.34)

T2DM, type 2 diabetes; BMI, body mass index; HOMA-IR, homeostasis model assessment insulin resistance.

### Complications

Adverse events reported at 6 years are shown in **Table 5**. There were no instances of postsurgical mortality or severe nutritional deficiency between the two groups; however, the incidence of marginal ulcers in the large gastric pouch group was significantly higher than that in the small gastric pouch group (23.7% vs. 6.4%, P=0.023). There were no significant differences in the prevalence of bleeding, gallstone diseases, intestinal obstruction, dumping syndrome or anemia (P>0.05). A large gastric pouch appeared to increase the risk for marginal ulcers (OR 1.13, 95% CI 1.08-1.34, P=0.047) compared with the risk associated with a small gastric pouch (**Table 4**).

**Table 5:** Complications at 6 years.

	Large gastric pouch (N=38)	Small gastric pouch (N=47)	P
Bleeding	2 (5.3%)	0	0.197
Gallstone diseases	1 (2.6%)	3 (6.4%)	0.625
Intestinal obstruction	1 (2.6%)	1 (2.1%)	1.000
Marginal ulcer	9 (23.7%)	3 (6.4%)	0.023
Dumping syndrome	2 (5.3%)	1 (2.1%)	0.584
Anaemia	3 (7.9%)	2 (4.3%)	0.652

## Discussion

Bariatric surgery has been widely performed worldwide over the past 60 years, and RYGB is considered to be the most effective treatment for obesity and obesity-related complications. However, the specific mechanism of RYGB is still unclear, especially the role played by the gastric pouch. At present, the existing literature simply illustrates the relationship between gastric pouch size and short-term weight loss outcome, and these results are inconsistent (11, 13).

The results of this study indicate that RYGB with a small gastric pouch achieves better weight loss than RYGB with a large gastric pouch among T2D patients with a BMI<35 kg/m2. Our current results demonstrated that the %EWL of the two groups was significantly different at 1, 3, and 6 years postoperation (P=0.041, P=0.037, and P=0.028, respectively). At the 6-year follow-up, there were significant differences in the changes in BMI and waist circumference between the two groups, and the small gastric pouch had significantly better outcomes than the large gastric pouch (P= 0.021 and P= 0.018). At 6 years postsurgery, the small gastric pouch group had significantly greater changes in BMI, %EWL, and waist circumference (-4.25 ± 0.51 kg/m2, 39.98 ± 3.29%, and -8.19 ± 1.46 cm, respectively) than the large gastric pouch group (-2.06 ± 0.48 kg/m2, 24.73 ± 3.15%, and -4.40 ± 1.12 cm, respectively) (P<0.05) (**Figure 3**). This finding also agrees with the study by Campos GM et al. (18), in which a larger pouch size was independently associated with poor weight loss after gastric bypass. Furthermore, a study by Riccioppo D et al. also showed that a small gastric pouch is associated with faster gastric emptying and leads to better long-term weight loss (19). In addition, Boerboom A et al. reported that an extended pouch (pouch length of 10 cm) improves midterm weight loss compared with the standard gastric pouch (pouch length of 5 cm) (20). In our study, long-term weight loss was relatively stable and was not as obvious as the early weight loss observed. Postoperative weight adjustment may be affected by physiological effects, eating habits, and self-monitoring behaviors, especially among patients who resume their original unhealthy eating patterns, consume more carbonated drinks, eat more fried food and consume high-sugar and high-fat diets postoperatively (21). Surprisingly, no differences were found in the levels of HDL, LDL, TG or TC at 6 years postsurgery. This is possibly explained by the complexity of the human body’s metabolic processes, which are influenced by genetics, certain diseases, drugs and/or environmental factors and lifestyles, which may affect lipid metabolism in some capacity, resulting in dyslipidemia (22).

Our research suggests that during the entire 6-year follow-up, the small gastric pouch allowed better glycemic control than the large gastric pouch (**Figures 4A, B**). The prevalence of T2D complete remission in the small gastric pouch group was significantly higher than that in the large gastric pouch group at 1, 3, and 6 years (56.6%, 49.0% and 46.8% vs. 33.3%, 27.5% and 23.7%, respectively). Significant differences were found in GHbA1c and FPG between the small gastric pouch group and the larger gastric pouch group at 6 years postoperatively (**Figures 4A, B**). The changes in GHbA1c and FPG plasma glucose levels in the smaller pouch size group were both significantly lower than those in the large gastric pouch group. In the long term, the small gastric pouch has a better postsurgical effect than the large gastric pouch and can help maintain better weight loss and glucose control. However, we observed a common phenomenon—with a prolonged follow-up time, both the T2D remission rate and weight loss showed a downward trend. This trend is in agreement with Mingrone’s findings, in which T2D remission was 75%, 37% and 25% at 2, 5 and 10 years after RYGB, respectively (23–25). A possible explanation for this phenomenon might be that as age increases, the function of pancreatic beta cells decreases each year (26). Pancreatic beta cell function is relatively insufficient among T2D patients, and insulin resistance is the main characteristic of this disease. The main reason for T2D relief after bariatric surgery is the improvement in insulin resistance, increasing insulin sensitivity; however, this improvement is limited (27, 28). In both groups, insulin resistance was improved, but the improvement in the small gastric pouch group was more significant; moreover, the T2D remission rate of the small gastric pouch group was correspondingly higher than that of the large gastric pouch group. In this respect, it is interesting to note that preoperative evaluation of islet cell function and insulin resistance is particularly important and has an important impact on the efficacy of the surgery among T2D patients. Restriction of food absorption and food intake is currently recognized mechanism for gastric bypass surgery, and small gastric pouch surgery can maximize satiety and reduce food intake. Small gastric pouch has greater advantages for insulin resistance and recovery of islet function.

The results of the multivariate regression analysis showed that a higher preoperative BMI and a small gastric pouch result in successful T2D remission, indicating that the size of the gastric pouch plays an important role in T2D remission. Similarly, the ABCD Diabetes Surgery Score (age, BMI, C-peptide, T2D duration) developed by Lee et al. also includes preoperative BMI (29). Obesity, especially central obesity, is closely related to insulin resistance and T2D in China (30). Bariatric surgery can significantly reduce insulin resistance, indicating why it is so effective for treating T2D (27). A small gastric pouch provides less ghrelin and superior restriction of food intake than a larger pouch in the long term (31). A randomized controlled clinical trial confirmed the theory that calorie restriction after RYGB accounts for the improvement in glycemic control (32).

Our results support the hypothesis that with the same Roux limb and biliopancreatic limb length, a small gastric pouch is associated with better outcomes than a large gastric pouch in terms of weight loss and glycemic control. Furthermore, our study shows that a large gastric pouch does not yield better weight loss and glycemic control and is associated with a higher incidence of marginal ulcers. Although the condition of these patients improved after regular proton pump inhibitor treatment, it is important to note that a smaller gastric pouch size is especially important for the low-BMI T2D population.

A number of limitations need to be noted regarding the present study. First, we did not specifically measure the exact size of the gastric pouch. Currently, there is no gold standard for the measurement of gastric pouch size. Most of the assessments of the gastric pouch are carried out by gastroscopy or computed tomography, and some scholars indirectly determine the size of the gastric pouch with a 40 French orogastric calibration tube or based on the total length of the stapler (9, 33, 34). In general, these methods indirectly reflect the size of the gastric pouch, and the stomach itself has a certain degree of ductility, which causes errors in the measurement. Additionally, our sample size was relatively small; thus, a randomized controlled trial with a large sample size is needed to further confirm our results.

## Conclusions

Overall, the small gastric pouch achieved better weight loss and glycemic control than the large gastric pouch at 6 years postoperation. A large gastric pouch resulted in a high prevalence of marginal ulcers (23.7% at 6 years postsurgery). Multiple regression analysis revealed that preoperative BMI and gastric pouch size could predict complete remission of T2D.

## Manuscript Formatting

### Headings

1. Small gastric pouch has better weight loss and glycemic control than large gastric pouch.
2. Small gastric pouch has better improvement of insulin resistance than large gastric pouch.
3. High preoperative BMI and a small gastric pouch are associated with better T2DM remission rates.
4. A large gastric pouch can lead to a higher incidence of marginal ulcers.

## Data Availability Statement

The raw data supporting the conclusions of this article will be made available by the authors, without undue reservation.

## Ethics Statement

The studies involving human participants were reviewed and approved by institutional review board of Third Xiangya Hospital. The patients/participants provided their written informed consent to participate in this study.

## Author Contributions

XG, formal analysis and writing-original draft. SD, data curation and methodology. GW, formal analysis. WL, writing-original draft. ZS, methodology. ZHS, data curation. SZ, supervision. LZ and PL, performed surgery, revised the draft, and conceptualization. All authors contributed to the article and approved the submitted version.

## Funding

This study was funded by the Wisdom Accumulation and Talent Cultivation Project of the Third Xiangya Hospital of Central South University [YX202106].

## Conflict of Interest

The authors declare that the research was conducted in the absence of any commercial or financial relationships that could be construed as a potential conflict of interest.

## Publisher’s Note

All claims expressed in this article are solely those of the authors and do not necessarily represent those of their affiliated organizations, or those of the publisher, the editors and the reviewers. Any product that may be evaluated in this article, or claim that may be made by its manufacturer, is not guaranteed or endorsed by the publisher.
